# Salivary levels of IgE and ECP in patients with recurrent aphthous stomatitis

**DOI:** 10.4317/jced.56254

**Published:** 2020-01-01

**Authors:** Leila Farhad-Mollashahi, Marieh Honarmand, Alireza Nakhaee, Shahram Kamalzadeh, Sanaz Amini

**Affiliations:** 1 Oral and Maxillofacial Diseases Research Center, Mashhad University of Medical Sciences, Mashhad, Iran; 2 Oral and Dental Disease Research Center, Department of Oral Medicine, School of Dentistry, Zahedan University of Medical Sciences, Zahedan, Iran; 3 Department of Biochemistry, School of Medicine, Zahedan University of Medical Sciences, Zahedan, Iran; 4 Dentist, Zahedan University of Medical Sciences, Zahedan, Iran

## Abstract

**Background:**

Recurrent aphthous stomatitis is a common oral mucosa disease, with no specific etiology. Atopy has been implicated in the development of this disease. In this study, the salivary levels of immunoglobulin E (IgE) and eosinophil cationic protein (ECP) were measured as allergy-related biomarkers in patients with aphthous stomatitis.

**Material and Methods:**

In this case-control study, non-stimulated saliva was collected from 85 participants and IgE and ECP were measured. Data were analyzed in SPSS 20 through the Mann-Whitney test, and *p*<0.05 was considered significant.

**Results:**

The salivary levels of IgE and ECP were 1.11±0.65 Iu/ml and 26.93±6.95 ng/ml in the case group and 0.73±0/39 Iu/ml and 21.97±6.72 ng/ml in the control group. There was a significant difference between the two groups in terms of salivary levels of IgE and ECP (*p*=0.001).

**Conclusions:**

The results showed that patients with oral aphthous had higher levels of salivary IgE and ECP than controls. Therefore, measurement of these biomarkers may be useful in the initial evaluation of patients with aphthous stomatitis.

** Key words:**Recurrent aphthous stomatitis, saliva, immunoglobulin E, eosinophil cationic protein.

## Introduction

Recurrent aphthous stomatitis (RAS) is a common oral mucosa disease (1-5) characterized by recurrent ulcers limited to the oral cavity without any symptoms of other diseases (4). Ulcers are very painful, interfere with eating, talking, and swallowing and affect the patients’ quality of life (6,7). The prevalence of aphthous stomatitis depends largely on the study population, although a prevalence of 5-25% has been reported (1,6). Studies have shown that oral aphthous is more prevalent in adult women, people under 40 years of old, whites, non-smokers, and those with high socioeconomic status (3).

RAS has an unknown etiology (1,8) and numerous factors have been implicated in its development including familial and genetic factors (9), nutritional, vitamin (10,11) and hematologic deficiencies (1,9), allergies (11,12), stress (8,13,14), immune reactions (8,9,11,15-18) and H. pylori infection (18).

Given the possible relationship of allergy and aphthous stomatitis, evaluation of allergy-related biomarkers, including eosinophil cationic protein (ECP) and immunoglobulin E (IgE), can be helpful in patients with aphthous stomatitis. ECP is an allergic inflammatory mediator, which is released from eosinophils and activated by IgE (19).

IgE is a type of five immunoglobulin classes in the body and plays an important role in the pathogenesis of allergic diseases. There are reports about changes in serum IgE levels in patients with aphthous stomatitis and increases in serum IgE levels in Behcet’s disease (12).

Almoznino *et al.* (12) reviewed the relationship between serum levels of IgE and demographic, clinical, and serological parameters of patients with aphthous stomatitis, and found a statistically significant relationship between the mean levels of IgE and female gender, age under 12 years, the onset and frequency of episodes of aphthous, and C-reactive protein level (CRP).

So far, no study has been conducted on the salivary levels of ECP in patients with aphthous stomatitis. In a study by Jang *et al.* (20) ECP was higher in people with allergic diseases than ECP in patients with non-allergic inflammatory diseases. Angelova Fischer *et al.* (21) observed an increase in serum levels of ECP in the acute phase of atopic dermatitis in comparison with the control group.

Since there may be an association between allergy and aphthous stomatitis and given the lack of studies on the allergy-related biomarkers in these patients, the present study aimed to evaluate these biomarkers in the patients’ saliva.

## Material and Methods

41 patients with recurrent aphthous stomatitis and 44 healthy individuals visiting the Zahedan Dental School were selected through convenience sampling method according to inclusion and exclusion criteria.

-Inclusion criterion in the case group

Occurrence of recurrent aphthous stomatitis at the visiting time.

Experience of at least three times of RAS per years.

Inclusion criterion in the control group

Healthy subjects matched with the case group in terms of age and gender.

Exclusion criteria in both groups

1. Any systemic disease, including allergic condition such as atopic dermatitis, allergic rhinitis, bronchial asthma.

2. Any drug consumption.

3. Pregnancy.

4. Occurrence of other oral mucosal diseases, including aphthous like ulcers.

5. Consumption of alcohol and tobacco products.

The study objectives were explained to all participants in the case and control groups and written consent was obtained from them. Ethical Committee of Zahedan University of medical science approved the present study (code: IR.ZAUMS.REC.1393.7013). The oral mucosa was examined using a disposable mirror under the dental unit light. RAS was diagnosed based on the following criteria:

Round or ovoid oral ulcers with circumscribed margins and a white or yellow pseudo-membrane surrounded by a red halo (11).

In addition, the complete medical history and demographic information of the patient were recorded in a questionnaire. Then non-stimulated saliva was collected from each person through the spitting method. To this end, all patients were asked to avoid eating, drinking, and brushing 90 minutes before sampling. All samples were collected between 9:00 and 12:00 AM, during which the patients were sat in a comforTable position and bent slightly forward. Every 1-2 min, they spitted their saliva in sterilized test tubes for 10 minutes. The test tubes were sealed with parafilm after the saliva collection, encoded, and immediately sent to the biochemistry laboratory. In the laboratory, the saliva was centrifuged for 10 min at 2000 rpm to separate the debris. The sample was transferred to a microtube using a micropipette. The microtube was also coded according to the test tube code and kept at -80°C for future testing (22).

The salivary levels of IgE and ECP were measured in the biochemistry laboratory through ELISA using the *Pi*shtaz Teb, Iran, and East Biopharm, china, kits, respectively.

-Data analysis

Data were analyzed using Mann-Whitney test in SPSS 20. *P*<0.05 was considered as the significance level.

## Results

In this study, 41 patients with recurrent aphthous stomatitis (25 males and 16 females) with an average age of 29 years were selected as the case group and 44 healthy subjects (21 males and 23 females) with an average age of 27 years were selected as the control group. There was no significant difference between the two groups in terms of age and gender ( Table 1).

**Table 1:**
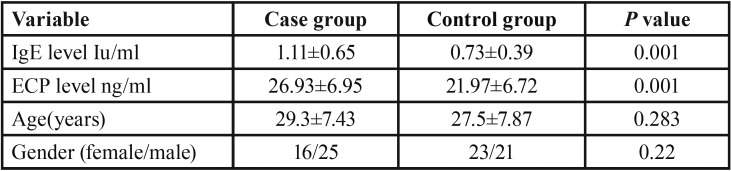
General baseline characteristics of the study groups.

Given the abnormal distribution of IgE and ECP levels, Mann-Whitney test was used, which revealed a significant relationship between recurrent aphthous stomatitis and increased salivary levels of IgE and ECP (*p*<0.0 5).

## Discussion

Recurrent aphthous stomatitis is a common oral mucosa disease (1-5) with still unknown etiology (1,8).

However, familial and genetic factors, immune system dysregulation, nutritional deficiencies, trauma, infectious agents (23) systemic diseases, stress and allergy to certain foods have been implicated in its occurrence (24). Several studies suggested the implication of atopy in the development of recurrent aphthous stomatitis (8,25,26). Exposure to some nutritional compounds such as chocolate, gluten, cow milk, and food colors can induce a pro-inflammatory cascade in the affected population. Dietary restriction has resulted in clinical improvement in some studies (8); however, Tarakji *et al.* (10) did not confirm the role of the diet in the occurrence of RAS. Given the possible role of allergy in the development of these ulcers, the present study aimed to investigate salivary IgE and ECP as allergy-related biomarkers. ECP is an allergic inflammatory mediator, which is released from eosinophils and activated by IgE. This leukocytic protein is toxic to neurons, and epithelial cell membrane and its levels do not directly correlate with the eosinophil count in peripheral blood (19).

Various studies have shown the association between ECP and allergic diseases (19-21,27-29).

Keles *et al.* (19) stated that serum levels of ECP and IgE are associated with the persistence of wheezing in people with asthma. Jang *et al.* (20) studied the levels of ECP in people with allergic diseases and showed that measurement of ECP is helpful in monitoring allergic diseases. This was confirmed in a study by Koh *et al.* (27)

Angelova-Fischer *et al.* (21) compared the serum levels of ECP and several other parameters in assessing the severity of atopic dermatitis in 21 patients in the acute phase and after complete remission. Their results indicated that the serum levels of these parameters were significantly higher in the acute phase of atopic dermatitis than in the control group and decreased along with symptoms improvement.

Schmekel *et al.* (28) attributed the increase in the salivary levels of ECP in asthmatic patients to the presence of eosinophils in the oral mucosa and salivary glands of asthmatic patients and the increased oral mucosal permeability due to harmful eosinophilic activity or increased ECP levels in the peripheral circulation. In a study by Lee *et al.* (29), restricted consumption of ready-made foods led to a reduction in serum ECP levels in children with atopic dermatitis and improved clinical symptoms.

IgE is one of the five isotypes of human immunoglobulins that plays a major role in the pathogenesis of several allergic diseases. Identification of IgE-coated lymphocytes in peripheral blood and RAS ulcers and increased number of mast cells in ulcers biopsy with symptoms of their activity and degranulation, indicating the role of these cells in the pathogenesis of RAS (12).

Almoznino *et al.* (12) showed an increase in serum IgE levels in these patients. Also, serum IgE levels were significantly associated with female sex, younger age, and early onset of RAS episodes. Fornasa and Gallina (25). examined the association between RAS and atopy in 39 patients with RAS in terms of their personal and familial history of atopy, serum IgE levels, skin prick test, skin patch test, and specific IgE Ab, and found clinical and laboratory signs of atopy in 27 patients. Ruan *et al.* (23) also reported that there is an association between RAS and atopy and showed that the serum levels of IgE were significantly higher in patients with RAS than in controls. In the present study, there was a significant difference in the salivary IgE between two groups, which was consistent with the mentioned researches. Our study is one of the few studies that investigated the salivary levels of IgE and ECP in patients with RAS; and due to the lack of similar study and difference of research methods, it was impossible to compare the results; this was one of the limitations of the present study.

Saliva is the result of serum outflow from the salivary glands supplying blood vessels, and diseases induced changes in the serum are reflected in the saliva; therefore, the use of saliva seems logical given its more comforTable, collection and maintenance, lower risk of HIV and hepatitis transmission, and cost-effectiveness (30).

## Conclusions

The results of this study showed that patients with oral aphthous had higher salivary levels of IgE and ECP than controls. Therefore, the levels of these biomarkers can be determined for the initial evaluation of patients with aphthous stomatitis.

## Suggestions

It is recommended to carry out a study with a larger sample size to investigate the relationship between the demographic, clinical, and serum parameters of the patients and the salivary and serum levels of these biomarkers.
